# Integrated bioinformatics analysis for identifying key genes and pathways in female and male patients with dilated cardiomyopathy

**DOI:** 10.1038/s41598-023-36117-0

**Published:** 2023-06-02

**Authors:** Min Zhang, Xinzhou Wang, Wenbo Chen, Wei Liu, Jile Xin, Debao Yang, Zhongyuan Zhang, Xiaoke Zheng

**Affiliations:** 1 College of Pharmacy, Henan University of Chinese Medicine, Zhengzhou, 450046 China; 2 The Second School of Clinical Medicine, Henan University of Chinese Medicine, Zhengzhou, 450002 China; 3 School of Medicine, Henan Polytechnic University, Jiaozuo, 454000 Henan China; 4 College of Life Sciences, Henan Agricultural University, Zhengzhou, 450002 China

**Keywords:** Gene regulatory networks, Biomarkers

## Abstract

Dilated cardiomyopathy (DCM) is a common cause of heart failure, and males are more likely to suffer from DCM than females. This research aimed at exploring possible DCM-associated genes and their latent regulatory effects in female and male patients. WGCNA analysis found that in the yellow module, 341 and 367 key DEGs were identified in females and males, respectively. A total of 22 hub genes in females and 17 hub genes in males were identified from the PPI networks of the key DEGs based on Metascape database. And twelve and eight potential TFs of the key DEGs were also identified in females and males, respectively. Eight miRNAs of 15 key DEGs were screened in both females and males, which may be differentially expressed in females and males. Dual-luciferase reporter assay demonstrated that miR-21-5P could directly target the key gene MATN2. Furthermore, Sex differences in KEGG pathways were identified. Both KOBAS and GSEA analysis identified 19 significantly enriched pathways related to immune response in both females and males, and the TGF-β signaling pathway was exclusively identified in males. Network pharmacology analysis revealed that seven key DEGs were potential targets for the treatment of DCM, of which the OLR1 gene was only identified in males, the expression levels of the seven genes were verified by RT-PCR. The above results could offer a novel understanding of sex differences in key genes and pathways in DCM progression.

## Introduction

Dilated cardiomyopathy (DCM) is characterized by ventricular enlargement and reduced myocardial systolic function, it is one of the common diseases that cause heart failure, arrhythmia, thromboembolism and sudden death^1^. The causes of DCM include genetic mutations, infections, exposure to toxins, inflammation, endocrine abnormalities, autoimmune or neuromuscular diseases^2^. There are sex differences in the incidence and prognosis of DCM^3^. Men are not only more likely to develop DCM but also exhibit worse prognosis as compared to women^4^. Male sex is recognized as an independent risk factor for poorer outcomes in DCM^5^, however, the molecular mechanism is still unclear. There are few studies about sex differences in pathogenesis or progression of DCM^2^.

The biomarkers that are being used for the etiological diagnosis of DCM include genetic markers and immune markers. The genetic markers include titin (TTN), lamin A/C (LMNA), β-myosin heavy chain (MYH7), cardiac troponin T (TNNT2), etc., whereas immune markers include anti-heart autoantibodies (AHAs)—the general term of antibodies against myocardial protein molecules produced by the body^6–8^. However, since sometimes elevated levels of biomarkers may be caused by various cardiovascular diseases^9^, diagnosis of DCM specifically is still challenging. Currently, both drug-based treatments, such as angiotensin-converting enzyme (ACE) inhibitors and β-blockers, and non-drug treatments, such as left ventricular assist device (LVAD) therapy and heart transplantation, can significantly improve the prognosis of DCM patients^7^. In spite of this, there is still a high mortality rate in DCM patients with heart failure^10^.

Weighted gene co‐expression network analysis (WGCNA) has been applied to multiple diseases to identify candidate biomarkers and therapeutic targets^11^. However, few studies have constructed co-expression modules for DCM^12–15^. In this study, gene expression profile data of female and male patients with DCM was analyzed to explore sex differences in the pathogenesis of DCM and to search for potential diagnostic biomarkers for DCM. After the identification of key differentially expressed genes (DEGs) in a major WGCNA module, hub genes in protein–protein interaction (PPI) networks, potential transcription factors (TFs) and microRNAs (miRNAs), and significantly enriched pathways were further analyzed to identify the differences between female and male patients. This research takes the lead in reporting sex differences in the key genes and pathways related to DCM base on bioinformatics methods. The pathogenesis of DCM is still unclear due to its heterogeneity, therefore, our findings would be beneficial for the diagnosis and prognosis of DCM.

## Materials and methods

### Dataset

Left ventricular transcriptome data of DCM patients and healthy donors were analyzed. Related gene expression data of DCM was downloaded from the Gene Expression Omnibus (GEO) database of the NCBI database (https://www.ncbi.nlm.nih.gov/). GSE141910 dataset contains the RNA-sequencing data of 332 samples originating from 100 DCM male patients (DCM-M), 66 DCM female patients (DCM-F), 77 healthy male donors (Control-M) and 89 healthy female donors (Control-F).

### WGCNA analysis

WGCNA R package was adopted for co-expression network construction. The weighted adjacency matrix was constructed as previously described^16^. Then, we transformed the weighted adjacency matrix into a topological overlap measure (TOM) matrix for estimating network connectivity^17^. Average linkage hierarchical clustering was carried out for constructing module dendrograms. To determine the importance of every module, Gene significance (GS) as well as module membership (MM) were calculated.

### DEGs and differentially expressed miRNAs (DEMs) analysis

The "limma" R package was utilized for screening DEGs as well as DEMs. The DEGs of male ("DCM-M" vs "Control-M") and female ("DCM-F" vs "Control-F") groups were screened from the GSE141910 dataset, whereas the GSE112556 dataset was analyzed to screen DEMs. The screening criteria of DEGs and DEMs were set as false discovery rate (FDR) ≤ 0.05 and |log_2_ fold change (FC)|≥ 1.

### PPI networks and potential TFs

The PPI networks and potential TFs were obtained by using Metascape database (http://metascape.org/gp/index.html#/main/step1)^18^. In this web-based algorithm, PPI network analysis was carried out by using STRING, BioGrid, OmniPath and InWeb_IM databases. And Molecular Complex Detection (MCODE) algorithm was used for identifying densely connected network components. Potential TFs were identified by using TRRUST database.

### Kyoto encyclopedia of gene genomes (KEGG) enrichment analysis

For identifying KEGG pathways^19,20^, KEGG orthology based annotation system (KOBAS) database was performed (http://kobas.cbi.pku.edu.cn/kobas3)^21^, following the criterion of corrected *P*-value < 0.05. Gene set enrichment analysis (GSEA) was conducted to analyze KEGG pathways by using an online platform (https://www.omicshare.com/tools), the criteria were set as |NES|> 1, *P* < 0.05 and FDR < 0.25.

### Network pharmacology analysis

The ingredients and targets of *Radix Astragali* were retrieved from the TCMSP database (https://old.tcmsp-e.com/index.php), and OB ≥ 30% and DL ≥ 0.18 were set as the criteria for screening ingredients. Subsequently, the gene symbols of targets were queried in the UniProt database (https://www.uniprot.org/). The intersection of the screened target genes and identified key DEGs were potential targets for treating DCM.

### Dual-luciferase reporter assays

The putative binding sequence and its mutant sequence were cloned into pmirGLO Dual-luciferase vectors (Promega). Then 293 T cells were transfected with the recombinant plasmids (MATN2 3'-UTR-WT/MATN2 3'-UTR-MUT) or empty plasmid, and hsa-miR-21-5p mimics or mimics-NC. Twenty-four hours later luciferase activity was measured using a dual-luciferase reporter assay kit (Promega).

### Animal experiments

The cTnT^R141W^ transgenic male mice were purchased from the Chinese Academy of Medical Science. The mice are maintained on a C57BL/6J genetic background and exhibit phenotypic characteristic of human DCM at 4 months of age^22^. Echocardiography showed that the left ventricular end-systole diameter (LVESD) of all the transgenic mice was greater than 2.64 mm. Mice at the ages of 4.5 months were sacrificed and the hearts were excised for RNA isolation. The animal experiment protocol of this study has been reviewed and approved by laboratory animal welfare and ethics committee of Henan University of Chinese Medicine (approval no. IACUC-202305020).

### Quantitative RT-PCR

Total RNA was extracted using Trizol reagent (Invitrogen, USA), and the cDNA was synthesized using a PrimeScriptTM RT reagent Kit (TaKaRa, China) according to the manufacturer's instructions. The gene expression levels of BNP, α-SMA, OLR1, CD40LG, CXCL10, CXCL11, COL1A1, DUOX2, and ESR1 were detected by the RT-PCR. GAPDH was used as an internal control. The primers are shown in a supplementary table (Table S1).

### Statistical analysis

Data were presented as mean ± SD. Statistical analyses were performed by one-way ANOVA and Tukey’s test. *P*-values < 0.05 were considered to be statistically different. *P*-values were adjusted for multiple testing to control the false discovery rate according to the method of Benjamini-Hochberg, FDR (q-value) < 0.05 was defined as significance cut-off.

## Results

### Dataset quality assessment

**Figure 1:**
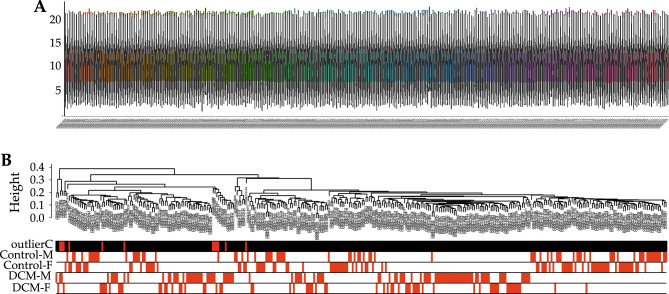
Normalization of gene expression data and samples clustering to detect outliers. (**A**) Gene expression profiles after normalization. (**B**) The sample dendrogram and trait indicator.

**Figure 2:**
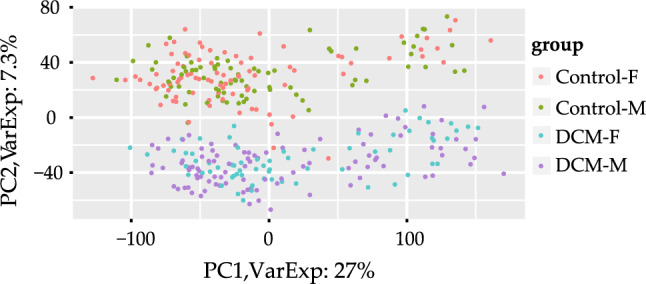
PCA plot of the normalized data for 320 samples.

After the expression data of DCM and control samples in dataset GSE141910 was normalized, the distribution of gene expression was observed to be consistent among 332 samples (Fig. 1A). Outlier samples (GSM4215867, GSM4215891, GSM4215904, GSM4215942, GSM4215956, GSM4215978, GSM4215980, GSM4215981, GSM4215993, GSM4216080, GSM4216124, GSM4216154) were identified among the 332 samples based on the connectivity. The 12 outlier samples were later removed from the 332 samples, and the remaining 320 samples (Fig. 1B) were further analyzed in the follow-up study. Principal component analysis (PCA) was then conducted, and 2D-PCA plots indicated a remarkable separation between DCM and control groups (Fig. 2).

### Identification of DEGs

Compared to the control group, 794 up-regulated and 322 down-regulated DEGs were identified in the female group, and 753 up-regulated and 363 down-regulated DEGs were identified in the male group.

### Identification of key DEGs in the yellow module

**Figure 3:**
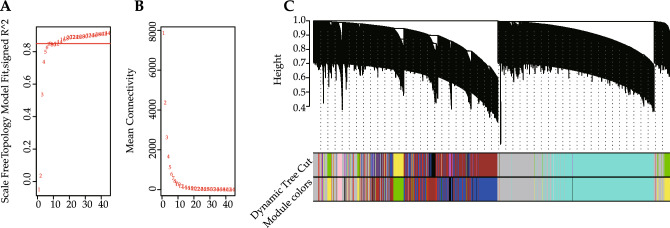
Construction of co-expression modules. (**A,B**) Analysis on the scale-free fit index and the mean connectivity. (**C**) Clustering dendrogram of genes in samples.

**Figure 4:**
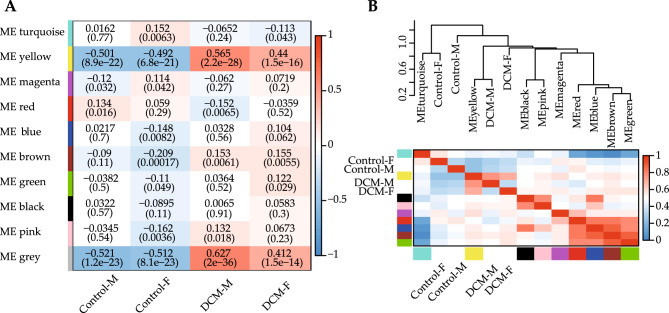
Identification of modules. (**A**) Module eigengene-trait associations. Each row indicates a module eigengene and each column represents a trait. Blue and red colors represent negative and positive correlations, respectively. Within each table cell, the upper values mean the degree of correlation, the lower values are the *P*-values. (**B**) Clustering dendrogram of module eigengene and heatmap of eigengene adjacencies.

WGCNA package of R was adopted for putting genes with similar expression patterns into modules, the power of β = 14 (scale-free R^2^ = 0.85) (Fig. 3A, B) was chosen to ensure a scale-free network. Altogether, ten co-expression modules (Fig. 3C) were identified based on the cluster dendrogram. The correlation between the four groups and ten modules was determined (Fig. 4A, B). Apart from the gray module (that contained unclustered genes), the yellow module correlated significantly with "DCM-M", "DCM-F", "Control-M" and "Control-F" groups, and this module showed the strongest correlation with "DCM-M" group (Fig. 4A). The module eigengene (ME) of the yellow module was negatively correlated with "Control-M" (−0.501) and "Control-F" (−0.492) groups, but positively correlated with "DCM-M" (0.565) and "DCM-F" (0.44) groups. Subsequently, the *P*-value < 0.05 of GS and MM was set as a threshold to screen key genes in the yellow module. Thus, there were 624 key genes identified in both "Control-F" and "DCM-F" groups, and 341 of the 624 genes were DEGs. There were 650 key genes identified in both "Control-M" and "DCM-M" groups, and 367 of the 650 genes were DEGs. Follow-up analyses were carried out for the 341 key DEGs in females and 367 key DEGs in males.

### PPI network analysis of the key DEGs

**Figure 5:**
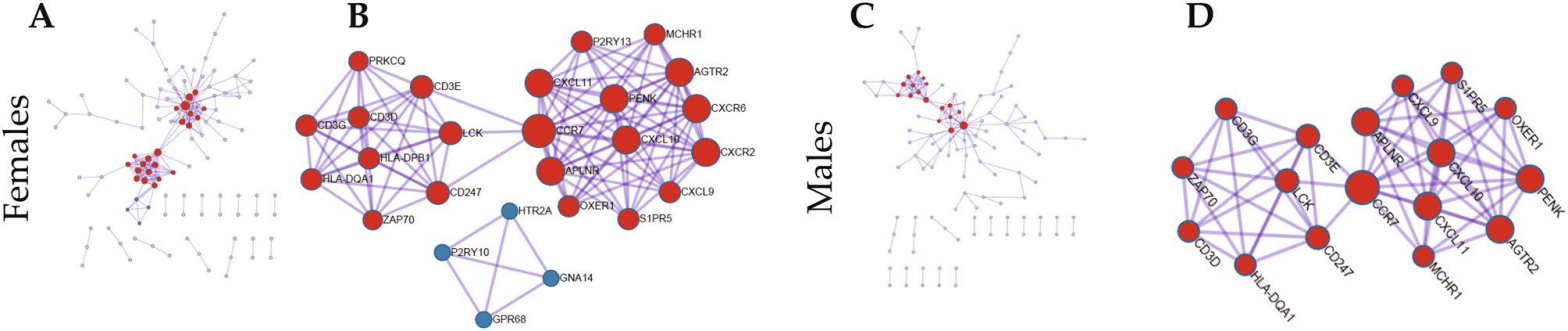
Protein–protein interaction (PPI) network analysis. PPI networks of the key differentially expressed genes (DEGs) in the yellow module of female (**A**) and male groups (**C**). The densely connected network components of female (**B**) and male groups (**D**) from the PPI networks.

PPI networks for the key DEGs in the yellow module were constructed (Fig. 5A, C). Based on this analysis, 22 genes in females and 17 genes in males from the densely connected network components were considered to be hub genes (Fig. 5B, D). Among these, the 17 hub genes (CCR7, CXCL11, PENK, CXCL10, CD3E, CD247, APLNR, AGTR2, CXCL9, OXER1, MCHR1, S1PR5, LCK, CD3G, ZAP70, CD3D and HLA-DQA1) were common in both female and male groups. Among the common hub genes, 7 (CD3E, CD247, LCK, CD3G, ZAP70, CD3D and HLA-DQA1) and 10 proteins (CXCL9, S1PR5, CCR7, MCHR1, OXER1, CXCL11, PENK, CXCL10, APLNR and AGTR2) respectively, interacted with each other in both females and males (Tables S2 and S3).

### Potential TFs of the key DEGs

**Figure 6:**
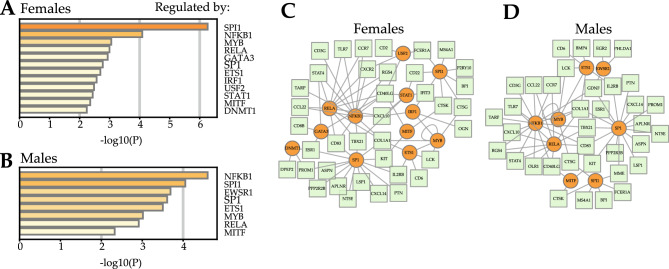
Transcription factors (TFs) analysis. Potential TFs of the key DEGs in the yellow module of female (**A**) and male groups (**B**). Regulatory networks of TFs and target genes in female (**C**) and male groups (**D**).

Twelve and eight potential TFs of the key DEGs were identified in the female and male group, respectively (Fig. 6A, B). Seven of these TFs (NFKB1, SPI1, SP1, ETS1, MYB, RELA and MITF) were identified in both groups. In both females and males, NFKB1 and RELA collectively targeted ten genes (CD40LG, CCR7, COL1A1, CXCL10, RGS4, CCL22, STAT4, CD83, TBX21 and TLR7); NFKB1, RELA and SP1 collectively targeted three genes (COL1A1, CD83 and TBX21); MYB and ETS1 collectively targeted three genes (COL1A1, LCK and MYB) (Fig. 6C, D).

### MiRNAs of the key DEGs

**Figure 7:**
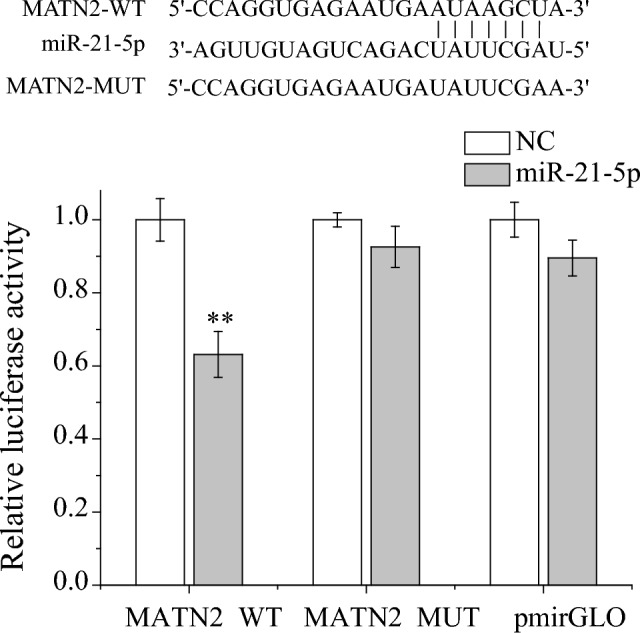
MATN2 was a direct target of miR-21-5p. (**A**) Schematic representation of the putative binding site between MATN2 and miR-21-5p. (**B**) MiR-21-5p significantly repressed luciferase activity in 293 T cells transfected with the MATN2 3'-UTR-WT plasmid. The error bars represent the standard deviation of three samples. **P < 0.01.

Based on the GSE112556 microarray data analysis, 13 DEMs were screened and identified in DCM patients. TargetScan, miRbase and DIANA-microT-CDS databases were used to predict target genes of miRNA separately, a total of 1217 target genes of the 13 DEMs were obtained from the three overlapped prediction results, and 15 of the 1217 genes were key DEGs in both females and males in the yellow module. As shown in Table 1, the 15 genes were the target genes of eight miRNAs, of which miR-9-3p targeted five genes (ITIH5, HAPLN1, NRK, CXCL14 and RYR3). In addition, miR-16-2-3p and miR-770-5p collectively targeted BCL11B, whereas miR-21-5p and miR-770-5p collectively targeted MATN2. To verify whether MATN2 is a direct target of miR-21-5p, we fused the MATN2 3'-UTR to a dual-luciferase reporter vector. The luciferase reporter assay results showed that compared to other groups, the relative luciferase activity was significantly repressed in the MATN2 3'-UTR-WT + miR-21-5p group (Fig. 7).

**Table 1:** Potential miRNAs of the 15 key DEGs in the yellow module.

Gene ID	Gene symbol	Upstream miRNAs of genes
80760	ITIH5	hsa-miR-9-3p
1404	HAPLN1	hsa-miR-9-3p
203447	NRK	hsa-miR-9-3p
9547	CXCL14	hsa-miR-9-3p
6263	RYR3	hsa-miR-9-3p
64919	BCL11B	hsa-miR-16-2-3p; hsa-miR-770-5p
5999	RGS4	hsa-miR-16-2-3p
10234	LRRC17	hsa-miR-21-3p
4147	MATN2	hsa-miR-21-5p; hsa-miR-770-5p
10125	RASGRP1	hsa-miR-21-5p
158158	RASEF	hsa-miR-144-3p
55512	SMPD3	hsa-miR-144-3p
116931	MED12L	hsa-miR-144-3p
117157	SH2D1B	hsa-miR-144-5p
8654	PDE5A	hsa-miR-382-5p

### KEGG pathways enriched by KOBAS and GSEA

**Figure 8:**
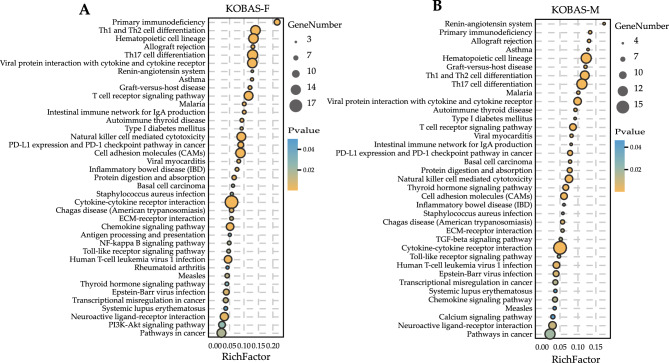
KEGG orthology based annotation system (KOBAS) was used to identify Kyoto encyclopedia of gene genomes (KEGG) pathways. The significantly enriched KEGG pathways of the key DEGs in female (**A**) and male groups (**B**). The sizes of the circle dots denote the numbers of enriched DEGs, larger circles indicate more enriched genes. The intensity of circle color means different *P*-values. RichFactor refers to the ratio of the enriched gene numbers to all annotated gene numbers in a pathway entry.

**Figure 9:**
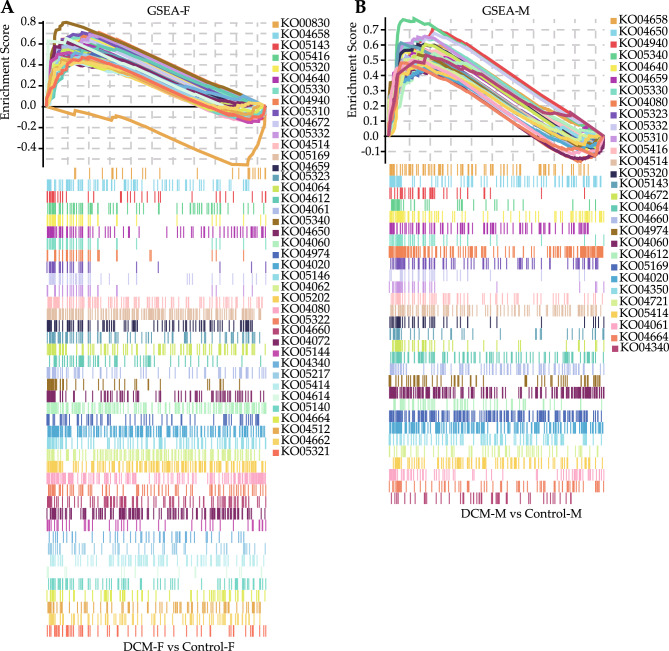
Gene set enrichment analysis (GSEA) was used to identify KEGG pathways. The significantly enriched KEGG pathways of the gene expression data of the dataset in female (**A**) and male groups (**B**).

**Figure 10:**
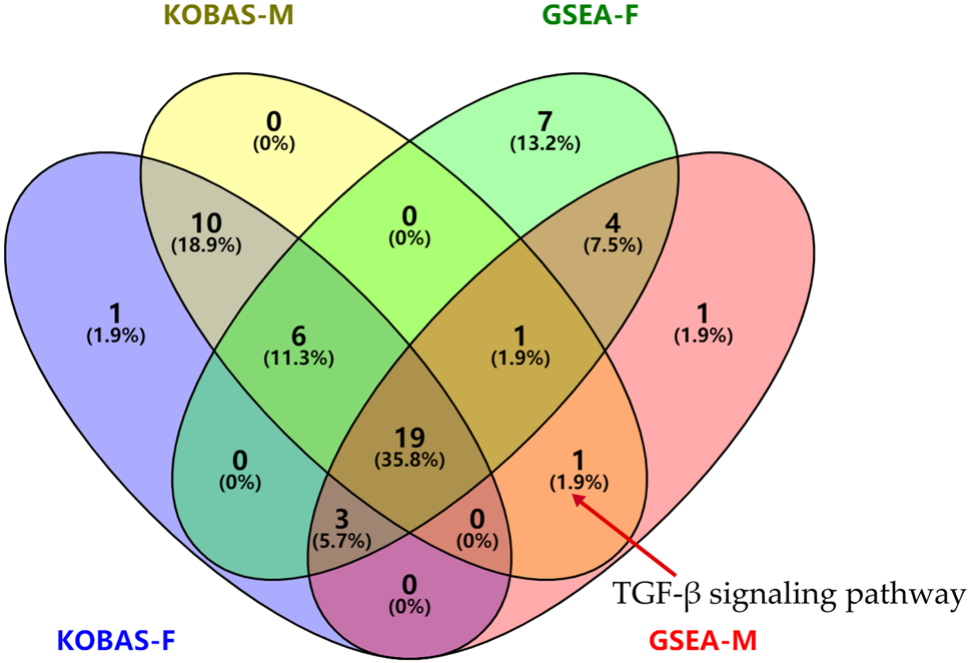
Comparative analysis of the number of KEGG pathways significantly enriched by KOBAS and GSEA.

KEGG pathways analysis for the key DEGs in the yellow module were conducted by the KOBAS web-based search tool. There were 39 and 37 significantly enriched pathways in the female and male groups, respectively (Fig. 8A, B). Out of these, 35 enriched pathways were common in the female and male groups. Four pathways including PI3K-Akt signaling pathway, NF-κB signaling pathway, antigen processing and presentation, rheumatoid arthritis were unique in the female group, two pathways including transforming growth factor (TGF)-β signaling pathway and calcium signaling pathway were observed to be unique in the male group. GSEA web-based search tool was also used to search enriched KEGG pathways by analyzing the gene expression profile data of the dataset (Fig. 9A, B). GSEA analysis indicated that the genes were significantly enriched in 40 pathways in the female group and 29 pathways in the male group (Tables S4 and S5), of which 13 and two pathways were specifically enriched in the female and male groups, respectively. Sex differences in KEGG pathways were analyzed by comparing the results of GSEA and KOBAS using a Venn diagram (Fig. 10). There were 19 common enriched pathways in the female and male groups, including Th17 cell differentiation, Th1 and Th2 cell differentiation, viral protein interaction with cytokine and cytokine receptor, T cell receptor signaling pathway, natural killer cell mediated cytotoxicity, autoimmune thyroid disease, viral myocarditis, primary immunodeficiency, allograft rejection, graft-versus-host disease, intestinal immune network for IgA production, Epstein-Barr virus infection, hematopoietic cell lineage, cytokine-cytokine receptor interaction, cell adhesion molecules (CAMs), asthma, protein digestion and absorption, neuroactive ligand-receptor interaction, type I diabetes mellitus. These enrichment terms are significantly associated with immune response. Interestingly, the TGF-β signaling pathway was exclusively identified in the male group, and it was not only enriched by GSEA but also by KOBAS. Five genes (GDF6, FMOD, BMP4, GREM1 and LEFTY2) in the TGF-β signaling pathway were found to have higher levels of expression among males compared with females (Fig. S1).

### Analysis of *Radix Astragali* target genes

**Figure 11:**
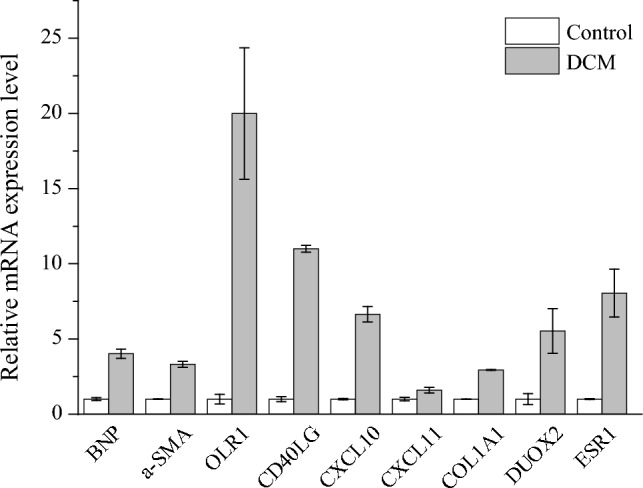
RT-PCR analysis of the mRNA expression levels of the seven target genes. BNP and α-SMA are marker genes.

The Network pharmacology method was adopted to screen target genes of *Radix Astragali* for treating DCM. Results showed that a total of seven target genes of *Radix Astragali* (Huangqi) were the key DEGs (Table S6). Six of these DEGs (ESR1, DUOX2, COL1A1, CXCL11, CXCL10 and CD40LG) were identified in both females and males, while OLR1 was only identified in males. Among these genes, CXCL11 and CXCL10 were the hub genes in the PPI networks. Except for DUOX2 and CXCL11, Other genes were targets of the identified TFs. KOBAS analysis indicated that the seven genes were significantly enriched in 40 pathways (Table S7), and these pathways are mainly related to immune response. The seven target genes were up-regulated in DCM, RT-PCR analysis verified that the expression levels of the seven genes were higher in the cTnT^R141W^ transgenic male mouse of DCM model (Fig. 11).

## Discussion

Cardiac fibrosis is a common pathological stage of a variety of cardiovascular diseases, and it is a mainly pathological change of DCM. Under pathological conditions, fibroblasts are transformed into myofibroblasts with a stronger ability to secrete extracellular matrix, the formation of myofibroblasts is a key step in the process of cardiac fibrosis^23^. Many factors are involved in regulating cardiac fibrosis, such as TGF-β, matrix metalloproteinases (MMPs), renin–angiotensin–aldosterone system (RAAS), inflammation, and non-coding RNAs (ncRNAs)^24^. The prevalence of cardiac fibrosis is higher in males than in females^25^. Studies have revealed that male mice with myocarditis have a much greater tendency to develop fibrosis and DCM than female mice^26^, more fibrosis has been also detected in male patients with myocarditis compared with female patients^27^. Therefore, Studying the genes involved in the regulation of cardiac fibrosis may be helpful to clarify the sex-based differences in the incidence or pathogenetic mechanisms of DCM.

In this study, the PPI networks and TFs of the key DEGs in females and males were analyzed by using Metascape database. These analyses could help to find some key factors involved in the regulation of DCM. We identified 17 hub genes from the PPI networks in both female and male groups. Several genes have been reported to be related to DCM or cardiac fibrosis. For example, HLA-DQ1 polymorphism is involved in the regulation of the immune response and may serve as genetic markers of idiopathic DCM^28^. The expression of CD3D and AGTR2 was increased in the heart following DCM^29,30^, and ventricular-specific expression of AGTR2 promotes the development of DCM and heart failure^31^. CXCL9 and CXCL10 are the main regulators of myocardial inflammatory cell migration and associated to the risk of DCM^32^. Apart from these, LCK, ZAP-70 and APLNR are also found to be associated with DCM^33,34^. TFs are proteins that play important roles in human diseases by binding to specific DNA sequence and regulating the transcription of related genes^35^. Seven common TFs were identified in female and male groups, previous studies indicate that four of these protein genes play important roles in DCM or cardiac fibrosis. Studies show that NFKB1 is involved in the process of cardiac remodeling, and the promoter polymorphism of the NFKB1 gene is associated with the risk of DCM^36,37^. It has been found that deletion of endothelial ETS1 in mice following angiotensin II infusion could alleviate cardiac fibrosis^38^. In addition, SP1 or MYB can mediate several miRNAs playing roles in regulating cardiac fibrosis^39–43^.

MiRNAs are a type of ncRNAs with a length of about 22 nucleotides, which negatively regulate gene expression through inhibiting mRNA translation or facilitating mRNA degradation, these molecules have been linked to multiple cardiovascular diseases^44–46^. A lot of evidence has shown that miRNAs are involved in regulating the process of cardiac fibrosis by regulating signal pathways or its target genes, thereby affecting the structure and function of the heart^47,48^. For example, studies have shown that miR-1, miR-133a, miR-208a/b and miR-499 participate in regulating cardiogenesis, heart function and pathology^49^. In our study, 13 DCM-related DEMs were obtained by analyzing the miRNA microarray data, and target genes of DEMs were predicted by using three databases. Previous reports have shown sex differences in cardiac miRNA expression^25^, thus miR-21, miR-144, miR-9-3p, miR-770-5p, miR-382-5p and miR-16-2-3p identified in this study may be expressed differentially in females and males. Fifteen target genes were identified in the key DEGs of both female and male groups based on WGCNA. Several studies have shown that some DEMs of the 15 target genes are involved in regulating cardiac fibrosis. For instance, miR-9 inhibits the proliferation of myocardial fibroblasts and the expression of collagen by inhibiting the expression of the target gene PDGFR-β or TGFBR2, and exerts an inhibitory effect on the fibrosis of myocardial fibroblasts^50,51^. MiR-144 has been found to have potent effects on reducing infarct size and border zone fibrosis in myocardial infarction model^52^. MiR-21 expression level is significantly increased in the failing heart, silencing of miR-21 can alleviate interstitial fibrosis and cardiac dysfunction by treating mice with a specific antagomir^53^. MiR-21 has also been revealed to be up-regulated in human left ventricular tissues of DCM^54^, which is in accordance with our analysis. The identified up-regulated target gene MATN2 was proved to be a direct target of miR-21-5p, it has been reported to be highly expressed in idiopathic DCM or heart failure^55,56^. More importantly, MATN2 could stimulate cardiomyocyte proliferation^57^. In addition, three target genes (ITIH5, NRK, PDE5A) in DCM and RGS4 in heart failure have been reported to be up-regulated, the expression trend of these genes is consistent with our analysis results^58–61^. These findings suggest that miRNAs and their corresponding target genes can serve as candidate diagnostic biomarkers or drug targets for the clinical treatment of cardiac fibrosis or DCM.

KOBAS and GSEA software were used to predict the significantly enriched KEGG pathways. Nineteen pathways, most of which are related to immune response, were identified in both females and males. It has been confirmed that the pathogenesis of DCM is related to autoimmune response^62^. Myocardial damage can be caused by abnormal immune responses, as it triggers inflammation and recruits immune cells to repair the myocardium, the cytokines released by immune cells such as Th17 and Th2 promote remodeling, collagen deposition and fibrosis, leading to heart enlargement, heart failure and arrhythmia^63,64^. The TGF-β signaling pathway was specifically identified in males. The expression of TGF-β has been observed to be increased in DCM patients^65^, studies have found that the cytokine TGF-β plays a vital role in the development of cardiac fibrosis and the regulation of immune response^66–68^. MiR-21 can activate TGF-β/Smad2/3 signaling pathway, cardiac fibrosis could be effectively inhibited by blocking this pathway^69,70^. Due to higher levels of gene expression in the TGF-β signaling pathway in males, thus, it is speculated that activation of TGF-β signaling pathway in males may promote cardiac fibrosis and DCM progression. Furthermore, estrogens have been found to possess antifibrotic effects, another reason that males are more likely to develop DCM than females may be due to the lack of estrogen protection^24,25,71^.

*Radix Astragali* is an important drug widely used in traditional Chinese medicine (TCM) prescriptions for the treatment of DCM, and its treatment effect is remarkable^72,73^. The seven key target genes of *Radix Astragali* were potential targets for treating DCM, especially the hub genes CXCL11 and CXCL10. The pathways significantly enriched by the seven genes further revealed that immune response may be involved in the progression of DCM. Some experimental results have demonstrated the mRNA or protein expression levels of the seven genes. Among the identified seven up-regulated genes, researches have shown that the levels of DUOX2, CXCL10 and CD40LG expression were significantly higher in patients with DCM^32,74,75^, and ESR1 and COL1A1 were substantially up-regulated in the mouse model of DCM^22,76^. In addition, the expression levels of CXCL11 and OLR1 have been proved to be elevated in heart failure patients^77,78^. We also verified that the expression levels of the seven genes were up-regulated in a mouse model of DCM by RT-PCR. These findings suggest that the seven genes are likely to play critical roles in the progression of DCM.

The expression trend of many key DEGs identified in this study is consistent with previously reported results, indicating that the database and our data analysis results are reliable.

## Conclusion

We analyzed gene expression profile data of female and male patients with DCM to explore sex differences in the pathogenesis of DCM and to reveal potential diagnostic biomarkers for this disease. And we identified the differences in key genes and pathways between female and male patients by using different analytical methods. This study can provide a reference for exploring diagnostic biomarkers of female and male DCM patients and their latent effects on disease development.

## Supplementary Information

Supplementary Figure S1.

Supplementary Table S1.

Supplementary Table S2.

Supplementary Table S3.

Supplementary Table S4.

Supplementary Table S5.

Supplementary Table S6.

Supplementary Table S7.

## Data Availability

All datasets are publicly available derived from NCBI databases. All data generated from the analysis process of this study are available from the corresponding author on reasonable request.
