# Supplementary material for: Integrated bioinformatics analysis for identifying key genes and pathways in female and male patients with dilated cardiomyopathy
Source: Sci Rep. 2023 Jun 2;13:8977. doi: 10.1038/s41598-023-36117-0 (PMC10238547; doi:10.1038/s41598-023-36117-0)
Supplement: Supplementary file 1 — Supplementary Figure S1. [file 41598_2023_36117_MOESM1_ESM.docx]

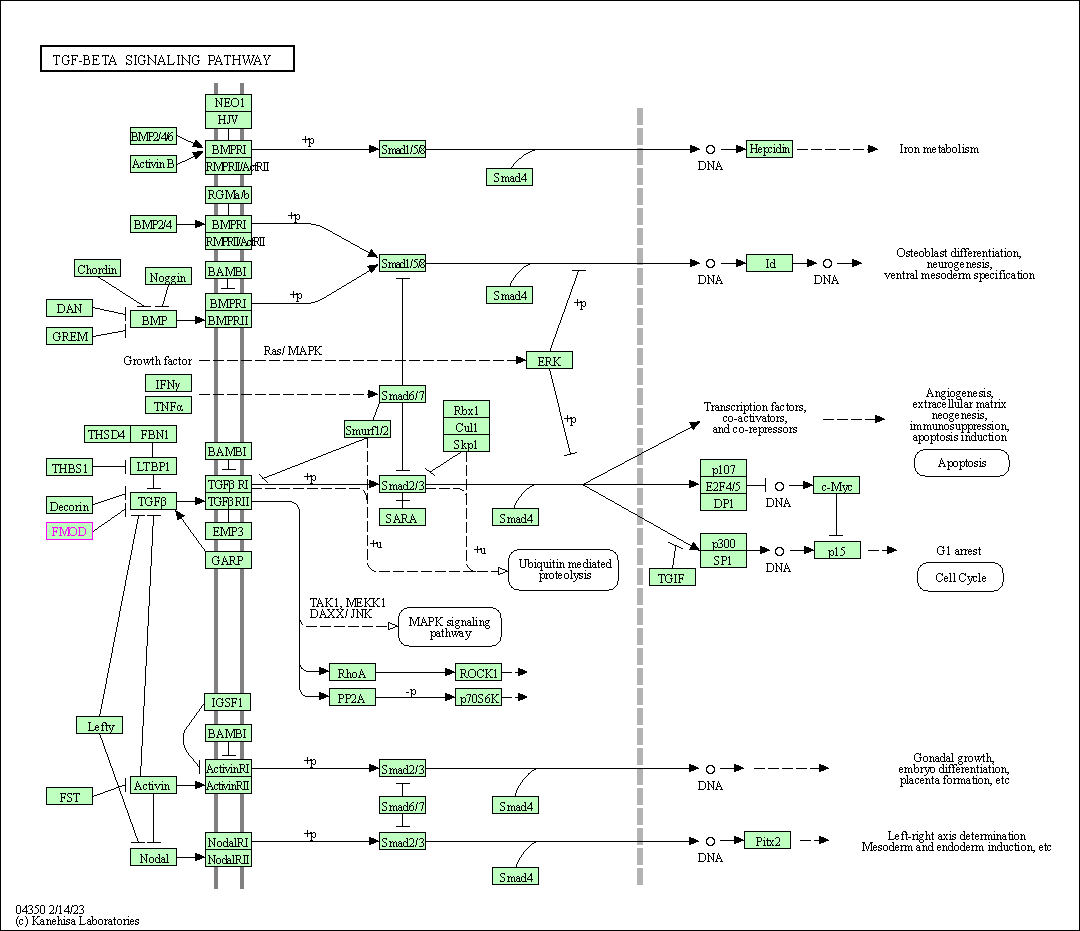


Fig. S1 TGF-β signaling pathway (map04350) of the KEGG pathway database. KEGG is developed by [Kanehisa Laboratories](https://www.kanehisa.jp/).
