# Supplementary figures and images for: Integrated bioinformatics analysis for identifying key genes and pathways in female and male patients with dilated cardiomyopathy
Source: Sci Rep. 2023 Jun 2;13:8977. doi: 10.1038/s41598-023-36117-0 (PMC10238547; doi:10.1038/s41598-023-36117-0)

**Table S1 The forward and reverse primers for RT-PCR.**


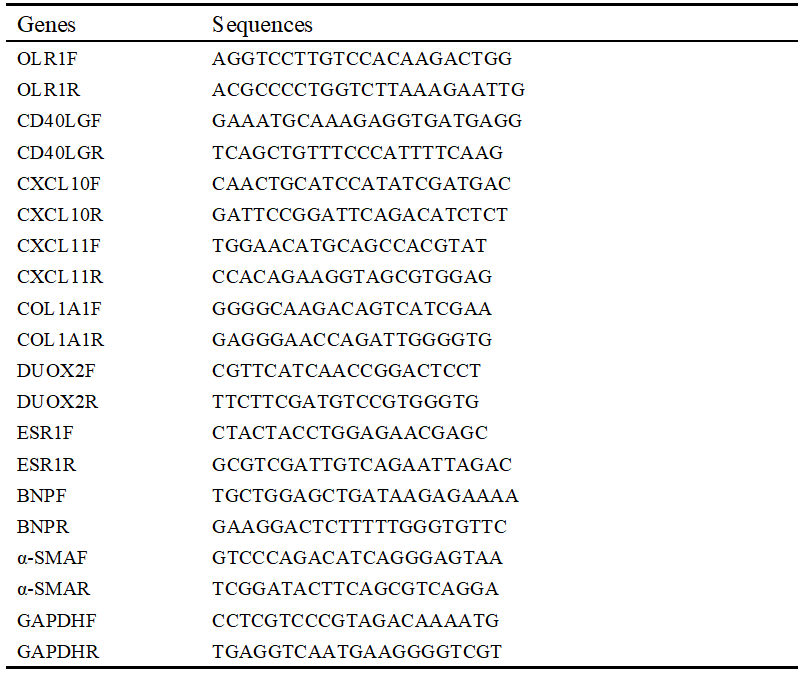

Supplement: Supplementary file 2 — Supplementary Table S1. [file 41598_2023_36117_MOESM2_ESM.docx]

**Table S2 The 22 hub genes in females and their interacting proteins.**


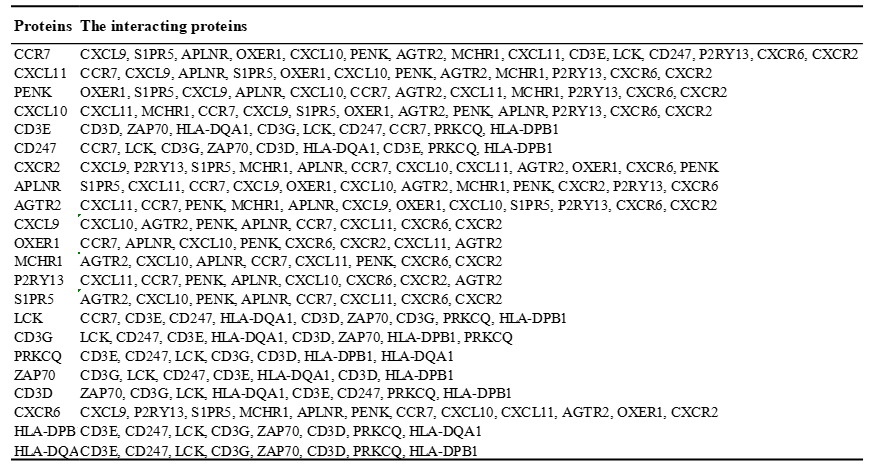

Supplement: Supplementary file 3 — Supplementary Table S2. [file 41598_2023_36117_MOESM3_ESM.docx]

**Table S3 The 17 hub genes in males and their interacting proteins.**


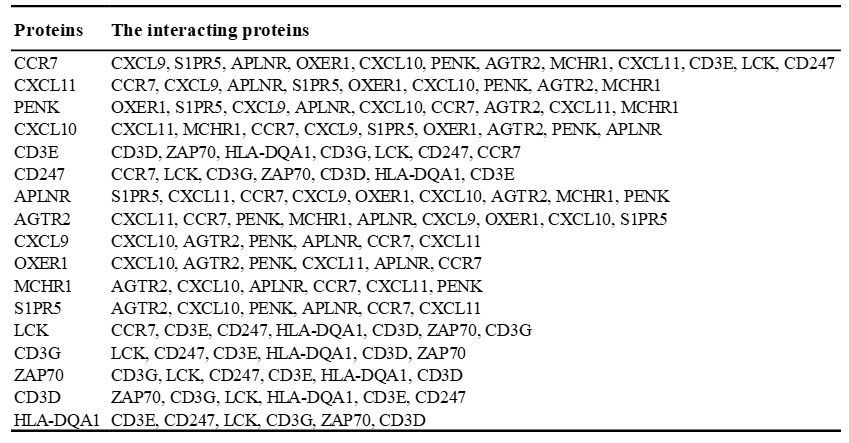

Supplement: Supplementary file 4 — Supplementary Table S3. [file 41598_2023_36117_MOESM4_ESM.docx]

**Table S4 GSEA analysis identified 40 significantly enriched pathways in females.**


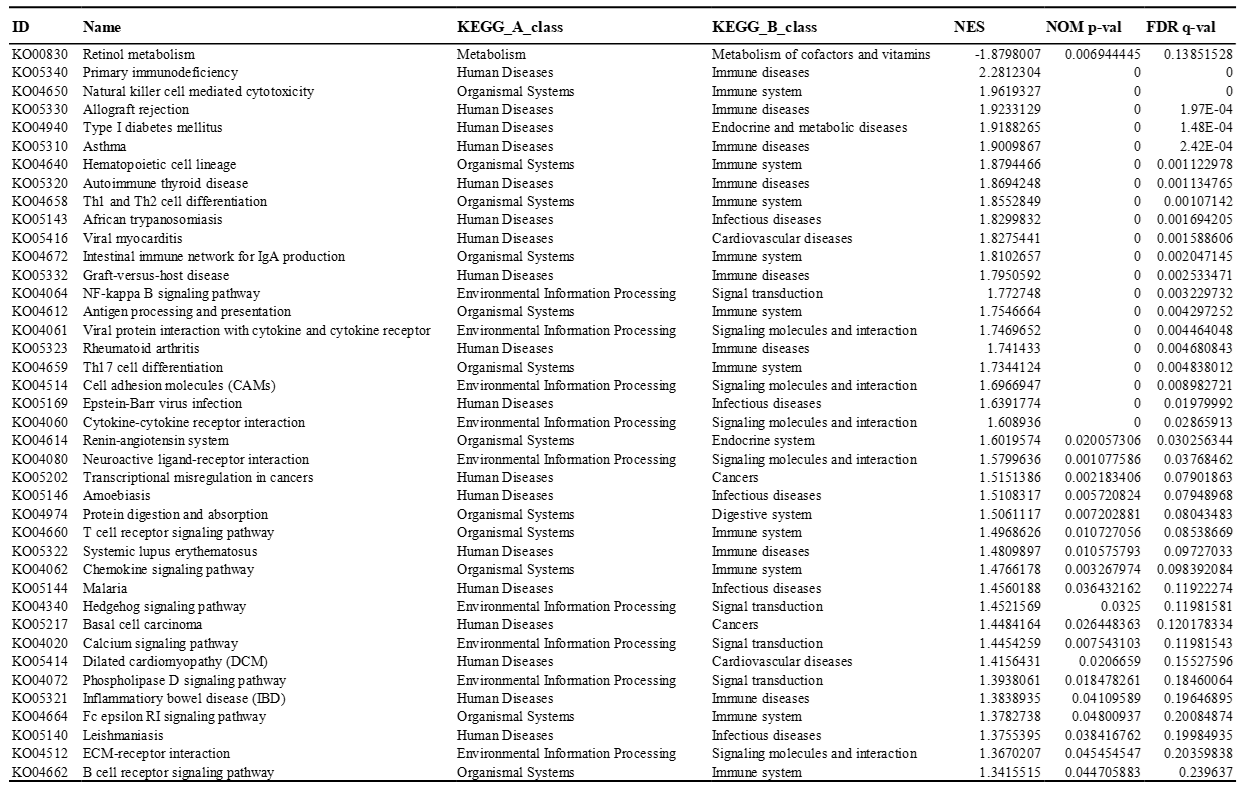

Supplement: Supplementary file 5 — Supplementary Table S4. [file 41598_2023_36117_MOESM5_ESM.docx]

**Table S5 GSEA analysis identified 29 significantly enriched pathways in males.**


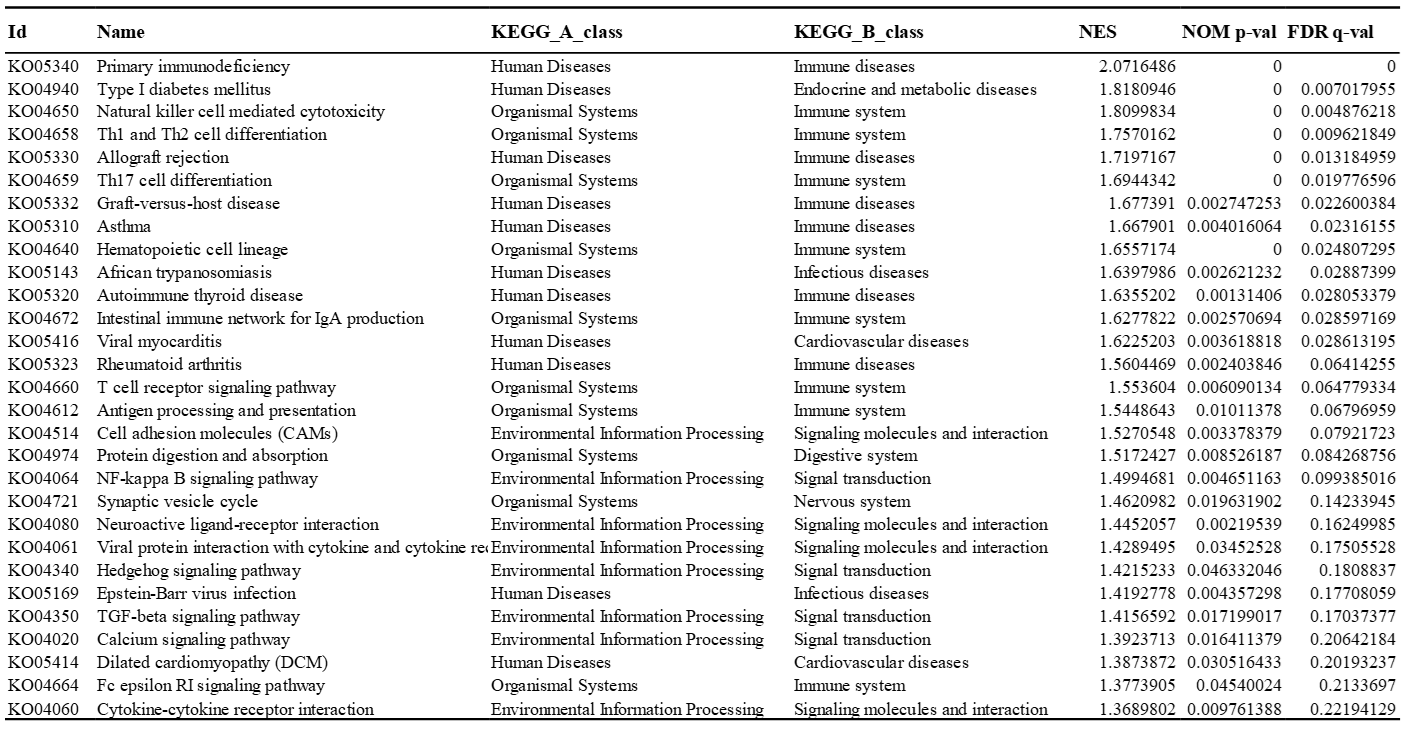

Supplement: Supplementary file 6 — Supplementary Table S5. [file 41598_2023_36117_MOESM6_ESM.docx]

**Table S6 Seven potential target genes for treating DCM.**


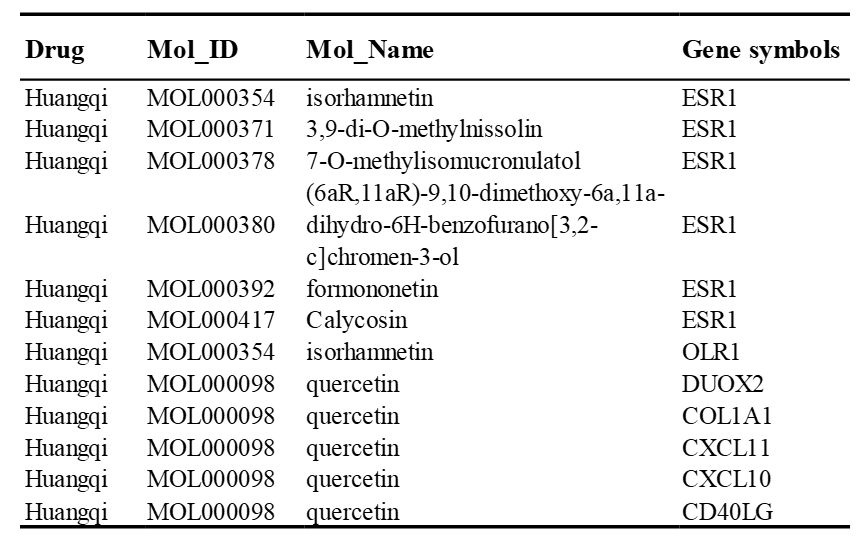

Supplement: Supplementary file 7 — Supplementary Table S6. [file 41598_2023_36117_MOESM7_ESM.docx]

**Table S7 Significantly enriched KEGG pathways of seven target genes.**


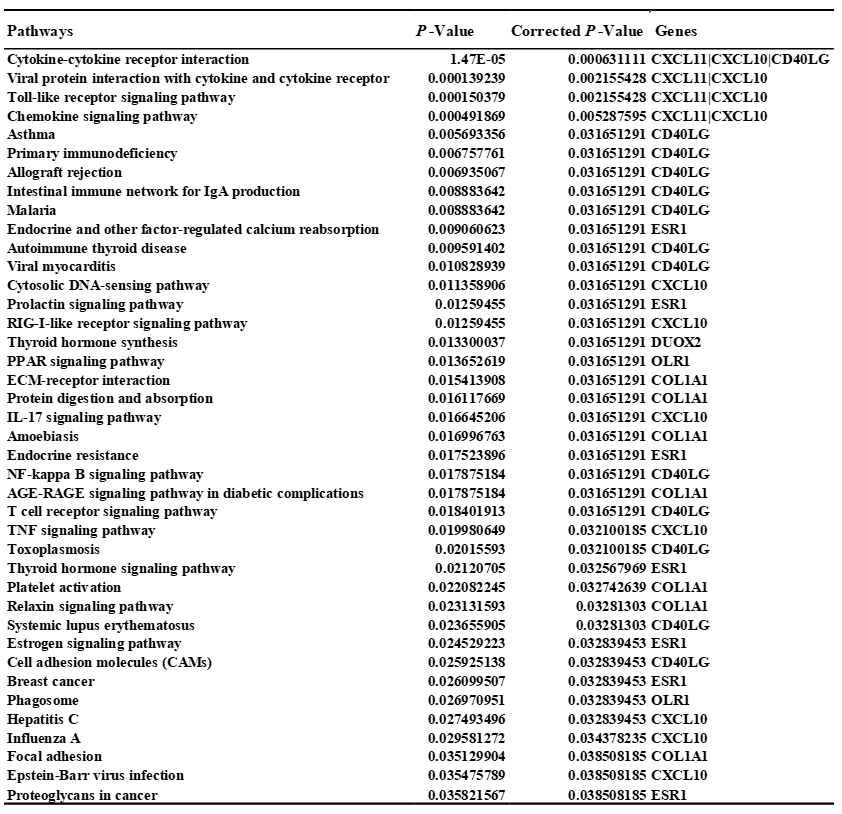

Supplement: Supplementary file 8 — Supplementary Table S7. [file 41598_2023_36117_MOESM8_ESM.docx]
